# *Aedes aegypti* (Diptera: Culicidae) Immune Responses with Different Feeding Regimes Following Infection by the Entomopathogenic Fungus *Metarhizium anisopliae*

**DOI:** 10.3390/insects11020095

**Published:** 2020-02-01

**Authors:** Sara Cabral, Adriano de Paula, Richard Samuels, Rodrigo da Fonseca, Simone Gomes, José Roberto Silva, Flávia Mury

**Affiliations:** 1 Laboratório Integrado de Bioquímica—Instituto de Biodiversidade e Sustentabilidade—NUPEM, Universidade Federal do Rio de Janeiro, Macaé, RJ 27965-045, Brazil; sara_z_c@hotmail.com (S.C.); betocfrio@gmail.com (J.R.S.); 2 Laboratório de Entomologia e Fitopatologia—CCTA, Universidade Estadual do Norte FluminenseDarcy Ribeiro, Campos dos Goytacazes, RJ 28013-603, Brazil; biodepaulaarquivo@gmail.com (A.d.P.); simoneazgomes@yahoo.com.br (S.G.); 3 Laboratório Integrado de Ciências Morfofuncionais—Instituto de Biodiversidade e Sustentabilidade –NUPEM, Universidade Federal do Rio de Janeiro, Macaé, RJ 27965-045, Brazil; rodrigo.nunes.da.fonseca@gmail.com; 4 Instituto Nacional de Ciência e Tecnologia em Entomologia Molecular—INCT-EM, Rio de Janeiro 21941-590, Brazil; 5 Laboratório Integrado de Biociências Translacionais—Instituto de Biodiversidade e Sustentabilidade—NUPEM, Universidade Federal do Rio de Janeiro, Macaé, RJ 27965-045, Brazil

**Keywords:** dengue, Zika, vector, immune response, gene expression

## Abstract

The mosquito *Aedes aegypti* is the most notorious vector of illness-causing viruses. The use of entomopathogenic fungi as bioinsecticides is a promising alternative for the development of novel mosquito control strategies. We investigate whether differences in immune responses could be responsible for modifications in survival rates of insects following different feeding regimes. Sucrose and blood-fed adult *A. aegypti* females were sprayed with *M. anisopliae* 1 × 10^6^ conidia mL^−1^, and after 48 h, the midgut and fat body were dissected. We used RT-qPCR to monitor the expression of Cactus and REL1 (Toll pathway), IMD, REL2, and Caspar (IMD pathway), STAT and PIAS (JAK-STAT pathway), as well as the expression of antimicrobial peptides (Defensin A, Attacin and Cecropin G). REL1 and REL2 expression in both the midgut and fat body were higher in blood-fed fungus-challenged *A. aegypti* than in sucrose-fed counterparts. Interestingly, infection of sucrose-fed insects induced Cactus expression in the fat body, a negative regulator of the Toll pathway. The IMD gene was upregulated in the fat body in response to fungal infection after a blood meal. Additionally, we observed the induction of antimicrobial peptides in the blood-fed fungus-challenged insects. This study suggests that blood-fed *A. aegypti* are less susceptible to fungal infection due to the rapid induction of Toll and IMD immune pathways.

## 1. Introduction

Many arbovirus vectors are hematophagous insects belonging to the order Diptera, such as mosquitoes, sandflies, black flies, and biting midges [1]. Mosquitoes transmit a significant proportion of these arboviruses. *Aedes aegypti*, for example, is responsible for the transmission of chikungunya, dengue fever, Rift Valley fever, yellow fever, Mayaro, and Zika virus, among others, and these viruses cause suffering and death worldwide [2,3]. Recent attention has focused on the Zika virus due to its association with congenital neurological disorders, such as microcephaly and Guillain–Barré syndrome [4,5,6].

Vector control using synthetic insecticides remains the main strategy to mitigate the spread of infectious diseases. However, chemical pesticides cause serious risks to human health, environmental pollution, and increased insecticide resistance [7]. To date, 57 countries have already documented the occurrence of *A. aegypti* and *A. albopictus* resistant to the most commonly used chemical insecticides [8]. The high cost and low sustainability of current vector control programs based predominantly on conventional insecticides and the lack of licensed vaccines for protection of the population against many of these viruses has stimulated increased interest in biological control [9,10,11].

Entomopathogenic fungi have been used in biological control programs against several insect pests [12]. Entomopathogenic fungi can control a range of stored-product and agricultural pests [13]. These fungi have gradually replaced conventional grain protectants during the past decade [14]. Entomopathogenic fungi, particularly those belonging to the genera *Metarhizium* and *Beauveria* have shown great promise as arthropod vector control tools [15].

Fungi can target all stages of the insects’ life cycle and are also able to infect insects with sucking mouthparts. Bacteria and viruses can only infect insects following ingestion and are therefore not capable of infecting adult mosquitoes. Entomopathogenic fungi can also cause infections following the ingestion of fungal propagules; however, the most common route of infection is via penetration of the host integument. The infection process initiates with the adhesion of spores onto the surface of the cuticle. Following adhesion, the spores germinate and form structures that aid in the penetration of the integument using a combination of physical and chemical (cuticle degrading enzymes) forces [16]. When the fungus breaches the integument, it then colonizes the hemocoel, rapidly spreading and proliferating, resulting in physical damage to the tissues and releasing toxins to suppress the insect immune system [17]. Depending on the level of inoculum and susceptibility of the host, death can occur within 24–48 h of infection. Upon the death of the insect, the fungus emerges from the host, covering the cadaver and producing large numbers of conidia, which can then infect other hosts [18].

Recent studies have shown that blood-fed *A. aegypti* females were less susceptible to *M. anisopliae* infections when compared to sucrose-fed females [19]. The reduced locomotion observed in recently blood-engorged mosquitoes reduced the contact time with fungus impregnated cloths, thus increasing survival rates when compared to sucrose-fed insects. Therefore, a greater understanding of the host–pathogen interactions is important, especially the little-studied role of the immune system in protecting *A. aegypti* against entomopathogenic fungi.

The immune system protects mosquitoes from invading pathogens and opportunistic microbes, as well as regulating the natural microbiota in the gut. The first obstacles to pathogen invasion are the physical barriers (integument and peritrophic membrane) and the first organ of contact for arboviruses is the mosquito midgut [20]. However, when a mosquito confronts a viral infection, cellular and humoral immune responses are activated and upregulated [21]. The innate form of the immune response is conserved across all organisms, including insects. Mosquitoes, like other insects, use the innate immune system to fight against pathogenic organisms [22]. The principal effectors of the cellular response are the hemocytes, which participate in phagocytosis, encapsulation, nodule formation, melanization, and tissue repair [23]. Humoral immunity mainly includes three major inducible responses, Toll, immune deficiency (IMD), Janus kinase (JAK)-signal transducer and activator of transcription (STAT) pathways, which express antimicrobial peptides (AMPs) via a signal transduction cascade, stimulating the production of melanin and reactive oxygen species (ROS) [24,25,26,27].

The insect antifungal immune mechanisms initially involve the recognition of fungal cell wall components [28], which then trigger downstream immune responses. However, many aspects of mosquito immune effectors against fungi are still unclear. Thioester-containing proteins (TEPs) are known to bind to foreign surface molecules and are thus considered critical for pathogen recognition [29]. TEPs have been shown to be involved in antifungal immunity in mosquitoes [30,31].

Toll is a major immune signaling pathway in insects and its antifungal characteristics have been documented [32]. Toll receptors are activated upon the binding of proteolytically cleaved Spätzle ligand, which leads to the activation of NF-kB factors, REL1 [33,34,35]. The effect of Cactus, (a negative regulator of REL1) on mosquito innate immunity was shown by decreased pathogen susceptibility and low levels of REL1 gene expression [35,36]. The Toll pathway can regulate the expression of several antimicrobial peptides, including DEF1 (defensin A and C), CEC1 (cecropin A), and GAM1 (gambicin) [37,38,39,40,41] The IMD pathway is another major signaling pathway that plays an essential role in mosquito immunity. This pathway has some downstream components overlapping with that of the Toll pathway in eliciting an immune response and has also been shown to confer immunity to fungal infection [30]. Like REL1 in the Toll pathway, REL2 plays a central role in IMD signaling as a downstream transcription factor [42]. The Caspar protein functions as a negative regulator of REL2, similar to the role of Cactus for REL1 [31,33]. The expressions of antimicrobial peptides such as DEF1, CEC1, and GAM1 are also regulated by the IMD pathway [37,39,40,43,44]. Another key player in the *A. aegypti* antifungal defense system is the JAK-STAT pathway, with PIAS acting as a negative regulator, inhibiting dimerized STAT proteins inside the nucleus [35,45]. In the *A. aegypti* cell line, activation of the JAK-STAT pathway by fungus leads to upregulation of GAM1 [39], which may contribute to antifungal defense.

In the current study, we investigated the immune responses of *A. aegypti* females to entomopathogenic fungi following infection of blood-fed or sucrose-fed mosquitoes, with the aim of understanding the influence of diet on the innate immune system and the subsequent development of fungal infection in this highly important vector.

## 2. Materials and Methods

### 2.1. Ethics Statement

All animal care and experimental protocols were conducted in accordance with the guidelines of the Committee for Evaluation of Animal Use for Research (Universidade Estadual do Norte Fluminense Darcy Ribeiro—UENF/CEUA) and the protocol was approved (register 248).

### 2.2. Animals

*Aedes aegypti* eggs were collected on the campus of the Universidade Estadual do Norte Fluminense using “ovitraps” [19]. Only F1 insects were used in all experiments. Larval eclosion was stimulated by total immersion of the eggs in water to which mouse food had been previously added (24 h) to reduce oxygen levels. Larvae were maintained in plastic trays (80 larvae per 100 mL) and fed on freshly ground mouse food (0.05 g per L) until reaching the pupal stage. Pupae were separated into water-filled beakers and transferred to cages before adult emergence. Adults were maintained in cages with wick feeders containing 10% sucrose. Two- to three-day-old female mosquitoes were used in all experimental assays. The mosquitoes were anesthetized using a stream of CO_2_ and females separated from males by anatomical differences in the antennae with the help of a magnifying glass.

### 2.3. Fungal Isolate and Preparation of Suspensions

The isolate of *M. anisopliae* used here was obtained from the collection at ESALQ (ESALQ818) in Piracicaba (São Paulo), which had been previously demonstrated to have high virulence against adult *A. aegypti* [46]. Fungi were cultured on Sabouraud dextrose agar (dextrose 10 g; peptone 2.5 g; yeast extract 2.5 g; agar 20 g in 1 L H_2_O) at 27 °C for 15 days before being used in experiments. Fungal suspensions were initially prepared in Tween 80 (0.05 % in sterile distilled water) and conidial concentration determined using a Neubauer hemocytometer. Conidial concentration was adjusted by serial dilution.

### 2.4. Infection of Mosquitoes with M. anisopliae and Survival Assays

Two- to three-day-old females were offered either 10% sucrose or mouse blood as described previously [47] before being exposed to *M. anisopliae*. Only mosquitoes that had taken a sucrose or blood meal (visibly engorged) were used in experiments. Immediately after feeding (time zero), the mosquitoes were sprayed with 1 mL of Tween 80 (0.05%) or *M. anisopliae* (1 × 10^6^ conidia mL^−1^) in Tween 80 using a Potter Tower (Burkart Ltd., Rickmansworth, UK) and then maintained in netting covered pots. This dose of fungus was used following preliminary experiments using higher doses (Supplementary Figure S1). The dose used here did not result in high levels of mortality during the first 48 h post-infection. All groups were offered filter paper discs soaked in 10% sucrose placed on the netting surface of the pots on a daily basis. Females at 48 h post-infection were dissected to obtain the midgut and fat body. All experiments were carried out three times with 45 insects per treatment: sucrose-fed, blood-fed, and controls (Tween only). The survival of *M. anisopliae* infected and control mosquitoes was monitored daily for up to 14 days. Tissue samples from these insects were used to detect gene expression of immune pathways.

### 2.5. RNA Extraction and cDNA Synthesis

For tissue-specific gene expression following fungal infection, midgut and abdominal fat body tissues were dissected from 15 mosquitoes per pool in a drop of 1× PBS at 48 h post-infection (PI). Samples were homogenized in TRIzol (Invitrogen, Carlsbad, CA, USA) and processed for Total RNA extraction according to the manufacturer’s instructions. Quality and concentration of the extracted RNA were assessed via NanoDrop (Thermo Scientific, Wilmington, DE, USA), and cDNA synthesis was conducted on normalized amounts of RNA using a High Capacity cDNA Reverse Transcription Kit (Thermo Fisher Scientific, Vilnius, Lithuania), according to the manufacturer’s instructions. RNA was treated with RNAse free TURBO™ DNAse (Thermo Fisher Scientific, Vilnius, Lithuania), and cDNA was synthesized from 1 μg of total RNA.

### 2.6. RT-qPCR Analysis

To detect Cactus and REL-1 (Toll pathway), IMD (IMD pathway), and STAT (JAK-STAT pathway) gene expression in the presence or absence of *M. anisopliae* infection, real-time PCR was carried out using cDNA preparations from each treatment group. The samples were assayed using an Applied Biosystems StepOne™ platform. Reactions were carried out in a total volume of 15 µL with 0.5 µM of primer (final concentration). Details of the specific primers for Cactus, REL-1, IMD, and STAT are shown in Table 1. The relative fold induction or repression in gene expression of experimental samples was determined and normalized using the ribosomal protein Rps17 (AAEL004175) [48] as a reference gene. The ribosomal protein Rps17 has been routinely used as a reference gene to assess *A. aegypti* transcript profiles [49,50]. Relative gene expression was assayed with the Comparative Ct (2^−ΔΔCt^) method and a validation assay was performed where serial dilutions are assayed for the target and reference gene. The standard curves were generated using five serial dilutions of the sample cDNA that reached exponential amplification at the earlier cycle [50].

### 2.7. Statistical Analysis

Comparisons between groups were carried out using non-paired Student’s *t*-tests with a 95% confidence interval or using one-way analysis of variance (ANOVA) followed by Newman–Keuls multiple comparison post-hoc test (GraphPad Prism) and, in this case, a difference of *p* < 0.05 was considered to be significant. The mosquito survival curves were analyzed by Kaplan–Meier pair-wise comparison [51]. Figures were generated using GRAPHPAD 6.0 software (California, USA). Error bars represent the means ± SD from three replicates.

## 3. Results

### 3.1. Effects of Fungal Infection on the Survival of A. aegypti Females

In order to evaluate the susceptibility of *A. aegypti* to *M. anisopliae* infection, both blood-fed and sucrose-fed mosquitoes were sprayed with conidial suspensions. To obtain relatively high levels of survival until dissection (48 h PI) for molecular analysis, a concentration of 1 × 10^6^ conidia mL^−1^ was chosen, since higher concentrations rapidly kill *A. aegypti* (Supplementary Figure S1), not allowing time for the detection of early immune responses.

**Figure 1:**
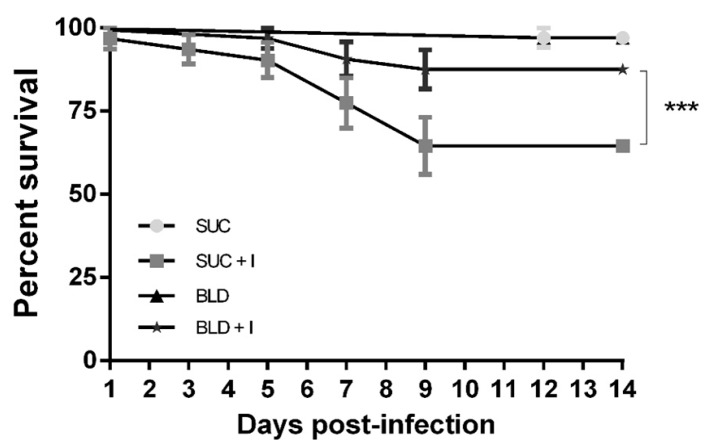
Susceptibility of *Aedes aegypti* adult female mosquitoes to fungal infection. SUC, sucrose-fed mosquitoes treated with Tween 80 (control); SUC + I, sucrose-fed mosquitoes infected with *M. anisopliae*; BLD, blood-fed mosquitoes treated with Tween 80 (control); BLD + I, blood-fed mosquitoes infected with *M. anisopliae.* Survival rates of *A. aegypti* (*n* = 45 per treatment) exposed to conidia of *M. anisopliae* ESALQ (ESALQ818) at a final dose of 10^6^ spores mL^−1^. The graph represents three independent experiments and data were analyzed with Kaplan–Meier survival analysis (GraphPad Prism 6 software). Error bars indicate the SEM. The fungus significantly increased SUC + I mortality compared to all other treatments (*** *p* < 0.001).

Mosquitoes exposed to the fungus showed reduced survival rates compared to their respective controls, independent of whether they were sugar-fed or blood-fed (Figure 1). However, a significant alteration in susceptibility was seen after blood feeding. Blood-fed mosquitoes were less susceptible to *M. anisopliae* infection than sugar-fed counterparts, with a significant difference seen between these two groups at the 95% level. All control treatments had high survival rates, with values >90% over the 14 days of evaluation. When using pair-wise comparisons of *M. anisopliae*-treated blood-fed and sucrose-fed females, the survival curves (Figure 1) were significantly different (see Supplementary Table S1).

### 3.2. The Immune Response of A. aegypti to Infection by M. anisopliae

**Figure 2:**
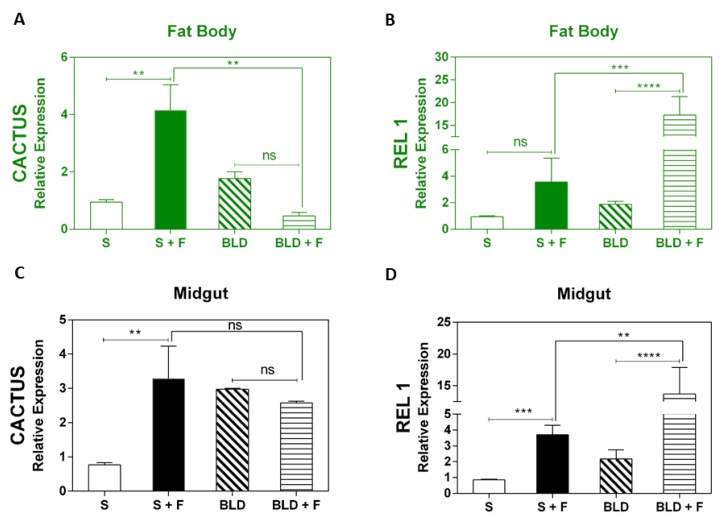
Expression analysis of TOLL pathway marker genes after exposure of *A. aegypti* to *M. anisopliae*. Gene expression analysis of Cactus and transcription factor REL1 in the midgut and fat body of sugar-fed or blood-fed insects at 48 h post-infection. The insects were fed with sucrose (10%) or mouse blood. After feeding, the insects were sprayed with *M. anisopliae.* Females at 48 h post fungal infection were dissected for total RNA extraction followed by synthesis of cDNA. cDNA was subsequently used to perform the RT-qPCR analysis. Each bar represents the results from a pool of 15 female mosquitoes. Results are the means of four independent experiments. The *p*-values from pair-wise comparisons (comparing Tween–treated to sugar-fed/blood-fed) were calculated using Student’s *t*-test and level of significance indicated for each bar (*: *p* < 0.05; b: ** *p* < 0.01; ***: *p* < 0.001; ****: *p* < 0.0001). Negative controls were treated with 0.05% aqueous Tween 80.

After confirming that blood feeding resulted in reduced susceptibility to fungal infection, we further investigated the role of canonical immune pathways during this process. Since Toll is the major immune pathway responsible for the antifungal response, we analyzed the Cactus and REL1 components in the midgut and fat body of infected mosquitoes following feeding on both sugar and blood. Expression analysis showed that the negative regulator Cactus was upregulated in the fat body of infected sugar-fed mosquitoes, while REL1 was not significantly affected (Figure 2A,B). However, in the fat body of blood-fed mosquitoes infected with the fungus, we observed a contrasting result. Cactus was not upregulated, but REL1 was significantly upregulated upon infection. In comparison, expression analysis in the midgut revealed that Cactus was significantly upregulated in fungus-infected sugar-fed mosquitoes. In the fungus-infected blood-fed mosquitoes, Cactus expression did not differ from uninfected control. However, the levels of Cactus in the blood-fed were not significantly different from the fungus-infected sugar-fed mosquitoes (Figure 2C). REL1 was upregulated in the midgut of both blood-fed and sugar-fed mosquitoes infected with fungus (Figure 2D).

**Figure 3:**
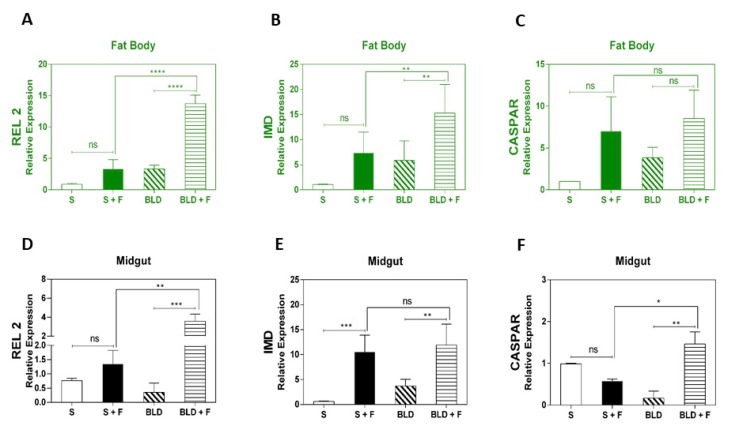
Expression analysis of IMD pathway marker genes after exposure of *A. aegypti* to *M. anisopliae*. Gene expression analysis of transcription factors REL2, IMD, and Caspar in the midgut and fat body of both sugar-fed and blood-fed insects at 48 h post-infection. The insects were fed with sucrose (10%) or mouse blood. Immediately after feeding, mosquitoes were sprayed with 0.05% Tween 80 solution, with or without *M. anisopliae.* Forty-eight hours post-infection, total RNA was extracted from mosquitoes, cDNA obtained, and RT-qPCR analyses subsequently performed. Each bar represents the results from a pool of 15 female mosquitoes. Results are the means of four independent experiments. The *p*-values from pair-wise comparisons (comparing Tween-treated to sugar-fed/blood-fed) were calculated using Student’s *t*-test and levels of significance were indicated for each bar (* *p* < 0.05; b: ** *p* < 0.01; *** *p* < 0.001; **** *p* < 0.0001). Negative controls were treated with 0.05% aqueous Tween 80.

The expression analyses of IMD marker components revealed that REL2 and IMD were upregulated in the fat body of fungus-infected blood-fed mosquitoes (Figure 3A,B). In the fungus-infected sugar-fed mosquitoes, the aforementioned genes showed no significant regulation upon infection (Figure 3A,B). Caspar, a negative modulator of the IMD pathway, showed no significant upregulation in the fat body of fungus-infected regardless of diet, even though there was a trend toward upregulation (Figure 3C). In the midgut, regulation tended to follow a similar pattern in relation to the fat body. Both REL2 and IMD were upregulated in blood-fed mosquitoes after infection (Figure 3D,E). IMD, in particular, was upregulated in the midgut of fungus-infected mosquitoes regardless of diet (Figure 3E). Caspar did not show any significant change in the midgut of fungus-infected sugar-fed, but in blood-fed mosquitoes, it was upregulated after infection (Figure 3F).

**Figure 4:**
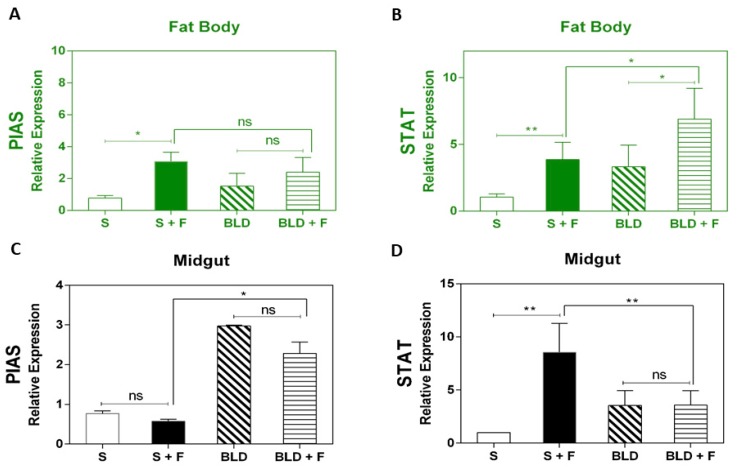
Expression analysis of the JAK-STAT pathway marker genes after exposure of *A. aegypti* to *M. anisopliae*. Gene expression analysis of *PIAS,* and signal transducer and activator of transcription STAT both in the midgut and fat body of sugar-fed and blood-fed mosquitoes at 48 h post infection. The insects were fed with either sucrose (10%) or mouse blood. Immediately after feeding, mosquitoes were sprayed with 0.05% Tween 80 with or without *M. anisopliae.* Forty-eight hours post-infection, total RNA was extracted from mosquitoes, cDNA obtained, and RT-qPCR analyses were subsequently performed. Each bar represents the results from a pool of 15 female mosquitoes. Results are the means of four independent experiments. The *p*-values from pair-wise comparisons (comparing Tween treated to sugar-fed/blood-fed) were calculated using Student’s *t*-test and levels of significance were indicated for each bar (* *p* < 0.05; ** *p* < 0.01; *** *p* < 0.001; **** *p* < 0.0001). Negative controls were treated with 0.05% aqueous Tween 80.

We then investigated the level of activation of the JAK-STAT pathway after infection in both sugar- and blood-fed mosquitoes. Expression analysis showed that STAT was upregulated in the fat body of fungus-infected mosquitoes regardless of the diet (Figure 4B). In the midgut, only sugar-fed mosquitoes showed STAT upregulation after fungal infection (Figure 4D). The negative modulator PIAS was significantly upregulated after infection in the fat body of sugar-fed mosquitoes. In fungus-infected blood-fed mosquitoes, PIAS did not differ from control uninfected blood-fed, but its expression was elevated in both and not significantly different from that of sugar-fed (Figure 4A). In the midgut, only blood-fed mosquitoes presented increased expression of PIAS regardless of infection.

### 3.3. Fungal Infection Induces Upregulation of Antimicrobial Peptides

**Figure 5:**
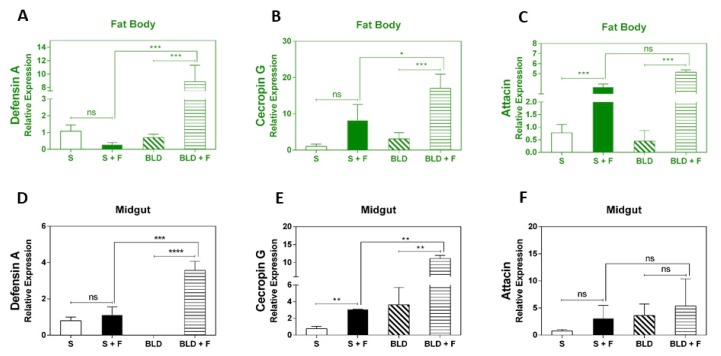
Expression analysis of antimicrobial peptides genes following infection with *M. anisopliae*. Gene expression analyses of defensin A, cecropin G and attacin in the midgut and fat body of sugar-fed or blood-fed at 48 h post-infection. The insects were fed with sucrose (10%) or mouse blood. Immediately after feeding, the mosquitoes were sprayed with 0.05% Tween 80 with or without *M. anisopliae.* Forty-eight hours post-infection, total RNA was extracted from mosquitoes, cDNA obtained, and RT-qPCR analyses were subsequently performed. Each bar represents the results from a pool of 15 female mosquitoes. The *p*-values from pair-wise comparisons (comparing Tween treated to sugar-fed/blood-fed) were calculated using Student’s *t*-test and levels of significance were indicated for each bar (* *p* < 0.05; b: ** *p* < 0.01; *** *p* < 0.001; **** *p* < 0.0001). Negative controls were treated with 0.05% aqueous Tween 80.

To assess the Toll pathway activation after the exposure of mosquitoes to *M. anisopliae*, we performed preliminary experiments by using RT-qPCR to monitor the transcript abundance of the Toll pathway marker genes: attacin, defensin A and cecropin G [38]. However, genes are regulated cooperatively or independently by the Toll and IMD pathways [35]. According to the pattern of expression of the different immune pathways observed, we investigated whether AMPs were also activated to combat fungal infection. We analyzed AMPs following *M. anisopliae* infection. Defensin A and cecropin G were significantly induced in the fat body of blood-fed insects following infection, whilst these two genes showed no significant upregulation in sugar-fed mosquitoes (Figure 5A,B). Attacin was induced independent of diet after fungal infection relative to controls (Figure 5D).

In the midgut, cecropin G was up-regulated regardless of diet following fungal infection, but its expression was more pronounced in the fungus-infected blood-fed mosquitoes (Figure 5D). Defensin A was only up-regulated in the fungus-infected blood-fed mosquitoes, while attacin showed no significant upregulation for either diet in tissues from infected mosquitoes (Figure 5E), even though these genes tend toward an increase in their expression.

## 4. Discussion

Immune responses in insects can be influenced by physiological state [52]. It is also possible that nutritional state can significantly affect insect susceptibility to infection by pathogens. Previous studies have shown that sucrose-fed *A. aegypti* females were more susceptible to fungal infection than blood-fed mosquitoes [19]. Herein, we provide molecular evidence confirming the influence of the feeding regime (blood or sucrose) on the differential susceptibility of *A. aegypti* to *M. anisopliae* infection and show that blood-fed mosquitoes are more efficient in mounting an immune response than their sucrose-fed counterparts.

The ability of arthropods to detect and avoid infection by entomopathogenic fungi can depend on species and developmental stage [17,53]. Ideally, entomopathogenic fungi should be virulent to their insect hosts, independent of host sex and physiological state.

Once mosquitoes take a blood meal, a cascade of events is activated to circumvent the stress resulting from this diet, mainly due to the presence of toxic heme and also due to the ingestion of potential pathogens during feeding [35]. Bonizzoni et al. [54] performed a global analysis of the changes in transcript accumulation following a pathogen free blood meal in *A. aegypti*. The investigation proved that blood feeding led to a reduction in the number of transcripts related to immunity at 5 h post-feeding. Their data were later corroborated by Bottino-Rojas et al. [55] after performing a transcriptome-wide analysis to investigate heme influence on *A. aegypti* cells. Overall, the components of the main immune pathways in the mosquito are downregulated by heme and this effect is reproduced after blood-feeding. This may explain why bacteria populations tend to increase in the midgut of blood-fed mosquitoes when compared to sucrose-fed counterparts. It is reasonable to assume that with the progress of the digestion, bacteria populations might be important to prime the immune system. In this regard, blood feeding can render the insect more efficient in eliciting an immune response, as confirmed in the present work. Yet Upton et al. [56] showed that blood feeding increased the transcript and protein levels of a leucin-rich repeat immune protein (LRIM) member, LRIM9, a known *Plasmodium berghei* antagonist. The increase in the protein levels was independent of bacterial presence. Immune activation may be a protective mechanism to combat potential invading microbes after the ingestion of a highly nutritional diet such as blood [57]. Moreover, it has been reported that pathogens present in the blood meal caused an enhanced production of immune effector molecules [55,58]. The interactions between insects and entomopathogenic fungi is complex [31,59]. In mosquitoes, antifungal immune responses are regulated by the Toll, IMD and Jak-STAT pathways [32,40].

The prominent activation of the *A. aegypti* Toll pathway in response to fungal, bacterial, and dengue virus infections suggests that this pathway controls multiple immune-related functions and defends the insect host against diverse pathogens [45]. The downstream component REL1 plays an essential role in the regulation of antifungal immune signaling through the Toll pathway [36].

The current study, which monitored Toll-related gene expression 48 h after fungal infection, demonstrated that REL1 levels are dietary specific, with significant upregulation both in the fat body and midgut of infected blood-fed *A. aegypti* mosquitoes (Figure 2B,D). Even though REL1 is significantly upregulated in the midgut of fungus-infected sugar-fed mosquitoes compared to control sugar-fed, its expression was significantly lower than in the fungus-infected blood-fed. The negative regulator of REL1, Cactus, was upregulated in the fat body of infected mosquitoes which had fed on sucrose (Figure 2A). Consequently, REL1 expression was not significantly altered upon fungal infection. In the midgut of fungus-infected sugar-fed mosquitoes, both Cactus and REL1 were significantly upregulated, suggesting that Toll is not strongly activated by fungal infection at this site. The increase in REL1 expression in both the fat body and midgut of blood-fed mosquitoes challenged with *M. anisopliae* provides evidence that activation of the Toll pathway can be influenced by the feeding regime. These results support the hypothesis that a blood meal activates the insect immune system after contact with the fungus, which enables the host to respond more efficiently to fungal infection, whilst sucrose-fed insects did not mount an efficient immune response and therefore higher mortality rates were observed in those insects.

Research has shown that the IMD pathway plays an important role in *A. aegypti* antifungal immune responses [32]. Comparative studies using different species of entomopathogenic fungi showed modulation of the IMD pathway transcription factor REL2 at the later stages of infection (6 days post-infection), with significant expression in the midgut and fat body tissues [31]. Here we investigate whether IMD pathway-controlled immune genes are responsive to different dietary resources using RT-qPCR to assess changes in transcript abundance of REL2, IMD, and Caspar during the early stages of infection, 48 h post-exposure to *M. anisopliae*. Our results showed a significant upregulation in REL2 expression in the fat body and midgut of infected mosquitoes after a blood meal (Figure 3A,D). An increase in IMD component expression was observed in both the fat body and midgut (Figure 3B,E). Garver and colleagues [60] showed that Caspar functions as a negative regulator of the IMD pathway and that the immune response against *Plasmodium* could be exaggerated by silencing the Caspar gene. Additionally, overexpression of the gene encoding REL2 transcription factor confers complete resistance against laboratory-cultured *P. falciparum* in *Anopheles gambiae* [37]. In our study, Caspar upregulation in the midgut of fungus-infected mosquitoes after a blood meal could be a strategy to avoid an exacerbated immune activation.

The immune signaling pathways protect mosquitoes from continuous exposure to invading pathogens and opportunistic microbes, as well as regulating the natural microbiota, for example, the gut flora [35]. In mosquitoes, the role of the midgut microbiota in influencing the IMD pathway after a blood meal was demonstrated through the induction of REL2 in the midgut and fat body [61]. In *A. aegypti*, ROS are continuously present in the midgut of sugar-fed female mosquitoes, but a blood meal immediately interrupts ROS production. This event occurred in parallel with an increase in gut bacterial levels [57]. In the absence of ROS, activation of immune pathways takes over this role in order to control the microbial population. It has been documented that fungal infection leads to an increase in the gut microbiota in mosquitos [30,62]. It is also possible that the gut microbiota plays a role in stimulating immune responses during infection of *A. aegypti* with *M. anisopliae*, although this was not investigated here. Wei et al. [62], for instance, showed that *Beauveria bassiana* infection led to an increase in the gut bacterial load, accelerating mosquito mortality in comparison to insects without microbiota, following antibiotic treatment. According to Oliveira et al. [57], bacterial load in the midgut of *A. aegypti* increases after a blood meal, in part as a consequence of diminished production of ROS. Heme may also be an important component contributing to increase bacterial load since it causes an immunosuppression in *A. aegypti* [55]. Yet Ramirez et al. [30], using different fungal species, showed that infection also led to an increase in the mosquito gut microbiota. However, this effect did not cause any difference in regard to survival. In addition, Ramirez et al. [31] showed compelling evidence that antibiotic treatment following fungal infection did not alter the quality of the immune response in comparison to those mosquitoes with normal microbiota. We therefore suggest that blood feeding provides the insects with better conditions to resist the fungal infection.

In addition to the IMD and Toll pathways, our study also showed that JAK-STAT was activated, specifically in the fat body of fungus-infected blood-fed mosquitoes but not in the midgut. A reasonable interpretation for this result could be in the route by which *M. anisopliae* invades their hosts. The conidia adhere to the cuticle and actively penetrate the integument [28]. Therefore, the fat body, which is situated just beneath the cuticle, quickly responds to the presence of the invading fungus. In the case of sucrose-fed mosquitoes, JAK-STAT was activated in the midgut, which could be an important regulator of microbiota proliferation. This could be the case in which the fungal infection might have led to an increase in the gut microbiota. The microbiota is naturally low in the gut of sucrose-fed mosquitoes [57] compared to blood-fed counterparts. So, this might help to explain why the sucrose-fed mosquitoes presented higher mortality rates than blood-fed counterparts, since a possible increase in microbiota after fungal infection might kill mosquitoes.

The induction of the immune signaling pathways may lead to the production of AMPs, which neutralize the invading pathogens. AMPs are small peptides that are mostly positively charged and are produced by the hemocytes, fat body, and midgut in response to signals received upon recognition of a pathogen [35,45]. The Toll pathway, activated in response to fungal infection, can trigger the transcriptional activation of defensin and attacin [38]. The IMD pathway plays a role regulating an important AMP, cecropin1 [43]. The expression of defensin and cecropin genes seen here also indicated a dietary specific dynamic. Both genes were induced in the fat body of fungus-infected blood-fed mosquitoes (Figure 5A,B). Attacin was up-regulated in the fat body regardless of the feeding regime. Furthermore, cecropin was also up-regulated in the midgut of infected mosquitoes regardless of feeding regime (Figure 5E). *A. aegypti* larvae were able to detect the presence of fungal pathogens almost immediately as AMP genes were elevated at 0 h post-infection, whilst defensin A and B remained elevated at 12 h post-infection, at which time many insects were dying [63]. Ramirez et al. [31] showed that the expression of IMD pathway-regulated antimicrobial peptides was tissue-specific and varied according to entomopathogenic fungal species. RNAi-based silencing of REL2 confirmed the importance of the IMD pathway since this led to an increase in fungal proliferation in *A. aegypti* infected with either *Beauveria bassiana* or *Isaria javanica*.

We demonstrate that the number of the immune-related genes differs between the tissues and different feeding regimes following fungal infection (Supplementary Table S2). These results suggest that the feeding regime has an important impact on the mosquito immune response. Blood feeding can bolster the mosquitoes’ ability to mount an immune response to combat fungal infection. The nutritional state is of great importance not only for immunity but also for many other metabolic issues. As discussed and reviewed by Rivera-Pérez et al. [64], non-energetic nutrients, including amino acids, might play many important roles for insect physiology. In terms of an immune response, Toll and IMD pathways seem to be of crucial importance in host defense. These findings are important in order to provide further information on the molecular interactions between entomopathogenic fungi and mosquitoes.

## 5. Conclusions

We confirmed that blood-fed females *A. aegypti* were less susceptible to *M. anisopliae* infection than sucrose-fed insects and that this could be due to higher expression of REL1 and IMD genes in the intestinal epithelium and fat body. In general, it was observed that blood-fed females infected with *M. anisopliae* expressed more defense genes when compared to sucrose-fed infected insects. This work provides insights into *Aedes* immune responses and these findings could impact the planning of biological control strategies.

## Figures and Tables

**Table 1:** Specifications of genes used for RT-qPCR analyses.

Gene	Primer sequence (5′–3′)	AAEL# ID (GenBank BankIt No.)
**Rps17**	FW GGGACAAATCGGCCAGGCTATC	AAEL009496
	RV TCGTGGACGCTTCTGCTTGTTG	
		
**AaREL1**	FW ATAGGCGAGATCAACATCAGCAGC	AAEL012164
	RV CGTTGCTGTTCCTGCTTCATATCG	
		
**AaREL2**	FW TTTGAATGTGCTGTTGGGTC	AAEL007624
	RV GAATGTTGTTTCCGTGCTTA	
		
**AaCACTUS**	FW AGACAGCCGCACCTTCGATTCC	AAEL000709
	RV CGCTTCGGTAGCCTCGTGGAT	
		
**AaIMD**	FW TGGTCAACCTGTTATGGCAA	AAEL010083
	RV GGGTTGACTTTGTCGTCGTT	
		
**AaCaspar**	FW CCTTTCTCGACCTACTTGCG	AAEL027860
	RV CGATCCTTATCAGTGCCGTT	
		
**AaPIAS**	FW GCTGCAACGCATGAAAACTA	AAEL000896
	RV CAGACGGGACAGTTCCAAGT	
		
**AaSTAT**	FW ACCGGACCTTCACCTTCTG	AAEL020559
	RV CCAGCTCACTGTTCGGAGAA	
		
**AaAttacin**	FW TTGGCAGGCACGGAATGTCTTG	AAEL003389
	RV TGTTGTCGGGACCGGGAAGTG	
		
**AaCecropin G**	FW TCACAAAGTTATTTCTCCTGATCG	AAEL015515
	RV GCTTTAGCCCCAGCTACAAC	
		
**AaDefensin A**	FW CTGCCGGAGGAAACCTATCAG	AAEL003841
	RV GCAATGCAATGAGCAGCACAAG	

## Supplementary Materials

The following are available online at https://www.mdpi.com/2075-4450/11/2/95/s1, Table S1: Kaplan-Meier analysis of all survival curves (all treatments), pair-wise comparisons of *M. anisopliae*-treated blood-fed and sucrose-fed female *A. aegypti* and comparisons of fungus-treated insects to their respective controls. Table S2: Summary of results from analysis of the immune related genes with different feeding regimes following fungal infection. Figure S1: Effects of *Metarhizium anisopliae* conidia on the survival rates of adults female *Aedes aegypti* mosquitoes exposed to different concentrations of the fungus.
