# Supplementary material for: Aedes aegypti (Diptera: Culicidae) Immune Responses with Different Feeding Regimes Following Infection by the Entomopathogenic Fungus Metarhizium anisopliae
Source: Insects. 2020 Feb 1;11(2):95. doi: 10.3390/insects11020095 (PMC7074208; doi:10.3390/insects11020095)
Supplement: Supplementary file 1 [file insects-11-00095-s001.pdf]

Table S1. Kaplan-Meier analysis of all survival curves (all treatments), pair-wise comparisons of *M. anisopliae*-treated blood-fed and sucrose-fed female *A. aegypti* and comparisons of fungus-treated insects to their respective controls.

**Treatments**	**All Treatments + Controls**	**Blood fed +Fungus *v* Sucrose fed + Fungus**	**Blood fed +Fungus *v* Blood fed controls**	**Sucrose fed +Fungus *v* Sucrose fed controls**
χ^2	26.22	24.07	2.19	14.14
***p* value**	< 0.0001	< 0.0001	0.1381	0.0002
**df**	3	1	1	1

Table S2. Summary of results from analysis of the immune related genes with different feeding regimes following fungal infection.

**Tissues**	**MIDGUT**		**FAT BODY**	
**Condition / Genes**	**Suc + F**	**Blood + F**	**Suc + F**	**Blood + F**
Cactus	+	-	+	-
REL 1	+	+	-	+
REL 2	-	+	-	+
IMD	+	+	-	+
CASPAR	-	+	-	-
PIAS	-	-	+	-
STAT	+	-	+	+
Defensin A	-	+	-	+
Cecropin G	+	+	-	+
Attacin	-	-	+	+
**Index**	**5**	**6**	**4**	**7**

Suc + F: sucrose fed and exposed to fungus; **Blood + F:** blood fed and exposed to fungus; **Index:** sum of positives.

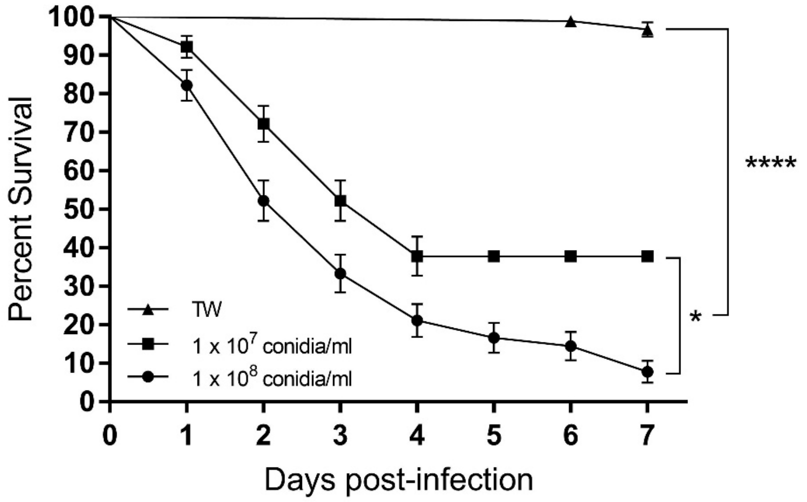

Figure S1. Effects of *Metarhizium anisopliae* conidia on the survival rates of adult female *Aedes aegypti* exposed to two concentrations of the fungus. TW – sucrose-fed adult female *A. aegypti* treated with Tween 80 (control); 1 × 10^7 – sucrose-fed adult female *A. aegypti* infected with *M. anisopliae* conidial suspensions (1 × 10^7 conidia/mL); 1 × 10^8 – sucrose-fed adult female *A. aegypti* and infected with *M. anisopliae* conidial suspensions (1×10^8 conidia/mL). n = 45 insects per treatment. The graph represents three independent experiments and data were analyzed with Kaplan-Meier survival analysis (GraphPad Prism 6 software). Error bars indicate the SEM. Conidia at both concentrations significantly increased mosquitoes mortality compared to each other and to the control treatment (*: $p < 0.05$; **** $p < 0.0001$).
